# Supplementary material for: Health workers’ perspectives on school-based mass drug administration control programs for soil-transmitted helminthiasis and schistosomiasis in Ogun State, Nigeria
Source: PLoS One. 2024 May 8;19(5):e0302509. doi: 10.1371/journal.pone.0302509 (PMC11078364; doi:10.1371/journal.pone.0302509)
Supplement: Attachment — Submitted filename: Comments by Reviewer_PONE-D-23-31424_R2.pdf [file pone.0302509.s004.pdf]

Comments by Reviewer: Dr Ogechukwu B. Aribodor

Manuscript Number: PONE-D-23-31424R2
Manuscript Title: Health Workers' Perspectives on School-Based Mass Drug Administration Control Programs for Soil-Transmitted Helminthiasis and Schistosomiasis in Ogun State, Nigeria.

General Comments:

I commend the authors for their efforts in significantly improving the manuscript. The revisions have enhanced the clarity and engagement of the content, providing valuable insights into the perspectives of health workers on school-based Mass Drug Administration programs for Soil-transmitted helminthiasis and Schistosomiasis in Ogun State, Nigeria. However, there are a few minor corrections that still need to be addressed for clarity and completeness.

1. Author summary: The health workers shared that working with **teachers' community leaders** and using various ways to communicate with people has helped reach out to communities. (Not clear, rephrase for more clarity).
2. Introduction: It would be helpful to include the commencement date (year) of the Mass Drug Administration (MDA) programs in Ogun State to provide context for readers.
3. Results: for the Tables especially Table 4 (Include footnotes for abbreviations like "NA" to ensure clarity for readers).
4. The acknowledgment stating, "We appreciate the Africa Research for Neglected Tropical Diseases, whose financial support under grant number SGPIV/0111/213 to FTA made this research possible," contradicts the information given earlier on how the study was funded (Financial Disclosure Section). Please review and ensure consistency in the acknowledgment section.

Thank you.
