# Supplementary material for: Health workers’ perspectives on school-based mass drug administration control programs for soil-transmitted helminthiasis and schistosomiasis in Ogun State, Nigeria
Source: PLoS One. 2024 May 8;19(5):e0302509. doi: 10.1371/journal.pone.0302509 (PMC11078364; doi:10.1371/journal.pone.0302509)
Supplement: Attachment — Submitted filename: PONE-D-23-31424_reviewer Comments 11 12 2023.docx [file pone.0302509.s001.docx]

**PNTD-D-23-01372_reviewer Comments**

**General Remarks:**

The manuscript titled "Health Workers' Perspectives on School-Based Mass Drug Administration Control Programs for Soil-Transmitted Helminths and Schistosomiasis in Ogun State, Nigeria" presents commendable efforts in addressing neglected tropical diseases (NTDs). However, **major** revisions are recommended to enhance the manuscript's overall quality and impact. These revisions should prioritize accuracy, clarity, and alignment with best practices in scientific reporting. The study's dedication is acknowledged, and the anticipation is for the revised version to significantly contribute to the scientific discourse in this field. Notably, the term "Soil-transmitted helminths" should be revised to accurately reflect the causative agents and the associated disease. The correct term is "Soil-transmitted helminthiasis" or "Soil-transmitted helminth infection." These revisions are crucial for maintaining scientific accuracy and should be incorporated consistently throughout the manuscript.

**Specific Comments:**

1. **Title:** Revise the title to accurately reflect the disease, using "Soil-Transmitted Helminthiasis" or "Soil-Transmitted Helminth Infection" instead of "Soil-Transmitted Helminths."
2. **Abstract:** Specify the study duration, providing a specific timeframe for the intervention and assessments. Clearly articulate the research problem and objectives in the introduction. Provide a brief yet comprehensive background on the relevance and impact of soil-transmitted helminths (STH) infection and schistosomiasis. Establish a stronger connection between the study's objectives and the broader context of NTDs.
3. **Methodology/Principal Findings:** Elaborate on the rationale for selecting the qualitative research approach. Clarify the criteria used for determining saturation in the absence of a formal sample size calculation. Elaborate on the process of pretesting interview topics and how reliability was ensured. Provide additional details on the content covered during the three-day training for data collectors. Consider providing more context on how discrepancies in coding were resolved among the research team. Clearly outline the selection criteria for health workers participating in the study. Provide additional details on the content of the semi-structured questionnaire. Include more information on the demographic characteristics of the study participants. Clarify the role of QRS NVivo 12 software in data analysis.
4. **Results:** Provide more context or examples to illustrate each thematic area in the results section. In other words, briefly explain the rationale for choosing the five thematic areas and their alignment with the research objectives. Provide additional information on the selection criteria for participants. Present participant demographics in more detail, such as years of experience, roles, etc. Define the demographic characteristics of participants before presenting thematic areas.

Ensure consistency in reporting results, including information on the number of participants in each thematic area.

5. **Discussion:** Start the discussion with a concise recapitulation of key findings.

Explicitly connect health workers' knowledge and engagement to program success.

Elaborate on potential challenges or limitations in forming and maintaining collaborations. Explore the role of technology or innovative communication methods in enhancing program effectiveness. Discuss the relative impact or severity of each identified barrier on program effectiveness. Discuss potential challenges or drawbacks of implementing the proposed recommendations. Consider prioritizing recommendations based on feasibility and impact. Emphasize the broader implications of the study findings for future research or policy changes.

Include the limitations of this study.

**Conclusion:** Provide a concise recapitulation of the main findings.

Elaborate on how collaborations can be sustained and expanded for long-term success. Briefly discuss specific strategies or approaches for improving communication in future programs. Provide more concrete details on how proposed solutions can be practically implemented or scaled up.

Expand on the specific components of the multifaceted approach and how they can be integrated into future programs.

**Overall:**

1. Ensure thorough proofreading for language and grammar.
2. Address all specific suggestions and clarifications outlined in each section.
3. Provide additional details, examples, or references where necessary to strengthen the manuscript's overall rigor.

Thank you.
