# Supplementary material for: Health workers’ perspectives on school-based mass drug administration control programs for soil-transmitted helminthiasis and schistosomiasis in Ogun State, Nigeria
Source: PLoS One. 2024 May 8;19(5):e0302509. doi: 10.1371/journal.pone.0302509 (PMC11078364; doi:10.1371/journal.pone.0302509)
Supplement: Attachment — Submitted filename: Reviewer 2_Comments.docx [file pone.0302509.s002.docx]

Reviewer #2:

**Dear Reviewer II,**

I would like to sincerely thank the reviewers for their insightful comments and suggestions regarding our manuscript titled "Health Workers' Perspectives on School-Based Mass Drug Administration Control Programs for Soil-Transmitted Helminthiasis and Schistosomiasis in Ogun State, Nigeria." We have carefully considered each comment and have made corresponding revisions to the manuscript. Below, we address each comment in detail and outline the changes we have made to improve our work.

1. Elaborate on the rationale for selecting the qualitative research approach.

Thank you for these comments. The rationale for the qualitative research approached has been stated in lines 124- 130.

*“A qualitative research approach was chosen for this study to delve deeply into the nuanced aspects of the MDA program. This approach was selected because it allows for a rich, detailed understanding of the complex realities and experiences of healthcare workers, educators, and other stakeholders involved in NTD control programs in Ogun State, Nigeria. Qualitative methods are particularly effective in capturing the diverse perspectives, attitudes, and social dynamics that quantitative methods might overlook, which is crucial for uncovering the underlying factors that influence the effectiveness of program implementation and addressing the barriers to success.”*

2. Clarify the criteria used for determining saturation in the absence of a formal sample size calculation.

Thank you. We used three criteria to determine when we had reached saturation: repetition of key themes, emergence of a coherent pattern in the data, and judgment that additional interviews would likely not yield new insights. This has been extensively explained in lines 182-188.

3. Elaborate on the process of pretesting interview topics and how reliability was ensured

We appreciate this comment. This information has been added in lines 200-211.

*“The* ***pre-test*** *of the interview topic involved a small group of individuals who had characteristics similar to the study population but were not part of the main study. Ensuring the reliability of the tool also encompassed refining the questions after pilot study to ensure clarity and the solicitation of the required information, validating findings with participants to ensure accurate reflection of their experiences and lastly engaging in peer debriefing”.*

4. Provide additional details on the content covered during the three-day training for data collectors.

Thank you for your comment. The content covered during the three-day training for the interviewers has been provided in lines 213-221.

*“The training introduced the research assistants to various qualitative data collection methods, such as IDIs, FGDs, and KIIs, each integral to comprehending the complex tapestry of human experiences. A significant portion of the training was devoted to ethical considerations, ensuring that the research assistants understood their role in conducting research with the utmost respect, dignity, and responsibility. Also, the training delved into ethnographic fieldwork, teaching research assistants to observe, participate, record, and reflect effectively. Role-playing exercises and field visit simulations were integral, sharpening their practical skills and preparing them for real-world complexities.”*

5. Clearly outline the selection criteria for health workers participating in the study.

Thank you. In lines 153-168, the new sentence reads;

*“Participants were carefully selected based on their roles within the health system, emphasizing direct responsibilities related to managing STH and SCH across different tiers of healthcare. The inclusion criteria focused on individuals actively involved in planning, executing, or evaluating control and prevention programs for NTDs in LGAs endemic with STH and SCH, representing a diverse mix of positions and expertise. The selection encompassed perspectives from policy and administration (DPH), local health programs (MoHs and LNTDs), and community interactions (CHEWs) to provide a comprehensive understanding of the challenges and strategies in managing NTDs, with attention to both experienced veterans and newer members.”*

6. Provide additional details on the content of the semi-structured questionnaire. Include more information on the demographic characteristics of the study participants.

Thank you for sharing your feedback. We have added a new table with the heading Key Elements of the interview guide in line 196 and the semi structured questionnaire used for the study has been added to the study Appendix.

***Table 1***

|  |  | *Key Elements* |
| --- | --- | --- |
| *FGDs* | *1* | *Community Illnesses & NTDs* |
|  | *2* | *Prevalence and Vulnerable Groups* |
|  | *3* | *Preventive Measures & Treatment* |
|  | *4* | *Community Efforts & Drug Availability* |
|  | *5* | *Program Awareness & Involvement* |
|  | *6* | *Communication & Barriers* |
|  | *7* | *Program Reception & Rumors* |
|  |  |  |
| *IDI/KIIs* | *1* | *Health Priorities & NTD Importance* |
|  | *2* | *NTD Occurrence & MDA Program Awareness* |
|  | *3* | *Communication & Barriers* |
|  | *4* | *Program Reception & Rumors* |
|  | *5* | *Data Management & Legislation* |
|  | *6* | *Involvement & Health Sector Priorities* |
|  | *7* | *Perceptions of NTD Programs* |
|  | *8* | *Integration with Health Programs* |
|  | *9* | *Program Evaluation & Stakeholder Involvement* |
|  | *10* | *Policies & Suggestions* |

Also, more information on the demographic characteristics of the study participants has been stated in Table 3 (290).

*Table 3: Socio-demographic characteristics of the study participants in the KIIs and FGDs.*

| *Role* | *Description* | *Frequency*  *(N=41)* | *Percentage (%)* |
| --- | --- | --- | --- |
| *CHEW* | *Gender* |  |  |
|  | *Male* | *23* | *71.9* |
|  | *Female* | *9* | *28.1* |
|  | *Age in years* |  |  |
|  | *30-35* | *14* | *43.8* |
|  | *36-40* | *5* | *15.6* |
|  | *41-45* | *12* | *37.5* |
|  | *46-50* | *1* | *3.1* |
|  | *Marital Status* |  |  |
|  | *Single* | *4* | *12.5* |
|  | *Married* | *21* | *65.6* |
|  | *Divorced* | *5* | *15.6* |
|  | *Widow* | *2* | *6.3* |
|  | *Religion* |  |  |
|  | *Christianity* | *19* | *59.4* |
|  | *Islam* | *13* | *40.6* |
|  | *Years of experience* |  |  |
|  | *3- 5* | *12* | *37.5* |
|  | *6-8* | *20* | *62.5* |
| *LNTD* | *Gender* |  |  |
|  | *Female* | *4* | *100* |
|  | *Age in years* |  |  |
|  | *36-40* | *1* | *25* |
|  | *41-45* | *3* | *75* |
|  | *Marital Status* |  |  |
|  | *Married* | *4* | *100* |
|  | *Religion* |  |  |
|  | *Christianity* | *3* | *75* |
|  | *Islam* | *1* | *25* |
|  | *Years of experience* |  |  |
|  | *3- 5* | *2* | *50* |
|  | *6-8* | *2* | *50* |
| *MOH* |  |  |  |
|  | *Gender* |  |  |
|  | *Male* | *2* | *50* |
|  | *Female* | *2* | *50* |
|  | *Age in years* |  |  |
|  | *41-45* | *1* | *25* |
|  | *46-50* | *2* | *25* |
|  | *51-55* | *1* | *50* |
|  | *Marital Status* |  |  |
|  | *Married* | *3* | *75* |
|  | *Divorced* | *1* | *25* |
|  | *Religion* |  |  |
|  | *Christianity* | *2* | *50* |
|  | *Islam* | *2* | *50* |
|  | *Years of experience* |  |  |
|  | *9-12* | *1* | *25* |
|  | *13-15* | *3* | *50* |
|  |  |  |  |
| *DoPH* | *Gender* |  |  |
|  | *Male* | *1* | *100* |
|  | *Age in years* |  |  |
|  | *46-50* | *1* | *100* |
|  | *Marital Status* |  |  |
|  | *Married* | *1* | *100* |
|  | *Religion* |  |  |
|  | *Christianity* | *1* | *100* |
|  | *Years of experience* |  |  |
|  | *13-15* | *1* | *100* |
|  | *Gender* |  |  |
|  | *Male* | *1* | *100* |
|  |  |  |  |

7. Clarify the role of QRS NVivo 12 software in data analysis

Thank you. The role has been clarified in lines 250-252.

*“QRS NVivo 12 software played a pivotal role in the data analysis process of the study, serving as a tool for managing, organizing, and analyzing the unstructured qualitative data collected from FGDs and KIIs”*
