# Supplementary material for: Health workers’ perspectives on school-based mass drug administration control programs for soil-transmitted helminthiasis and schistosomiasis in Ogun State, Nigeria
Source: PLoS One. 2024 May 8;19(5):e0302509. doi: 10.1371/journal.pone.0302509 (PMC11078364; doi:10.1371/journal.pone.0302509)
Supplement: Attachment — Submitted filename: Response to Reviewer_Akinsolu.docx [file pone.0302509.s003.docx]

**PNTD-D-23-01372_reviewer Comments**

I would like to sincerely thank the reviewers for their insightful comments and suggestions regarding our manuscript titled "Health Workers' Perspectives on School-Based Mass Drug Administration Control Programs for Soil-Transmitted Helminthiasis and Schistosomiasis in Ogun State, Nigeria." We have carefully considered each comment and have made corresponding revisions to the manuscript. Below, we address each comment in detail and outline the changes we have made to improve our work.

Specific Comments:

1. Most references cited in the first paragraph of the introduction, are not appropriate and authoritative for the associated quotes. Same applies to Reference 9. Authors should recheck. Citation should be from WHO, Hotez and a couple of others who gave this foundational metrics.

Thank you for your recommendation. The references have been updated appropriately as suggested.

2. Also, the definition of MDA here should be clearer than this; the concept of delivery of at risk population without prior diagnosis is very important, same as the need to mention the medicines used for the diseases, same as the strategy of using drug distributors, also the recommended guidelines based on endemicity, and the objective of the entire process, which is to meet 75% therapeutic coverage to interrupt transmission cycle.

Thank you for your observation. The definition of MDA has been modified; it is written as thus.

*“The 2030 NTD Roadmap has a strategic focus on controlling and eliminating NTDs, which has a significant portion of its success in implementing integrated mass drug administration (MDA) programs [9, 10] as the primary approach for controlling and potentially eliminating NTDs. MDA involves periodically administering safe, effective, low-cost drugs to at-risk populations until disease-specific targets are met [11].”* (See lines 80-84)

3. Same applies to Reference 9. Authors should recheck. Citation should be from WHO, Hotez and a couple of others who gave these foundational metrics.

Thank you. The sentence has been supported with other references as recommended.

4. Authors only cited Ref 15. Since they mentioned “certain studies”. They in fact need to further strengthen this claim with a couple more references. If they have local programmatic data/evidence in the study area they have chosen that would also be more relevant.

Thank you for your observation. We added the following sentences to more explanation in the paragraph.

*“As recommended by the WHO, a single dose of albendazole (400 mg) or mebendazole (500 mg) in MDA to treat STH is efficient [10, 14]”* (See lines 88-89)

In line 93

*“Notable, the MDA program initiative has played a pivotal role in alleviating the burden of SCH and STH [15, 16]. In an endemic area, the WHO recommends that at least 75% of school-age children (SAC) should receive MDA [17, 18]”* (See lines 89-91)

Additionally, more references have been added to strengthen our claim in the statement.

*Griswold E, Eigege A, Adelamo S, Mancha B, Kenrick N, Sambo Y, et al. Impact of three to five rounds of mass drug administration on schistosomiasis and soil-transmitted helminths in school-aged children in north-central Nigeria. J The American Journal of Tropical Medicine Hygiene. 2022;107(1):132.*

*Agboraw, E., Sosu, F., Dean, L. et al. Factors influencing mass drug administration adherence and community drug distributor opportunity costs in Liberia: a mixed-methods approach. Parasites Vectors* ***14****, 557 (2021).* [*https://doi.org/10.1186/s13071-021-05058-w*](https://doi.org/10.1186/s13071-021-05058-w)

*Makaula, P., Kayuni, S. A., Mamba, K. C., Bongololo, G., Funsanani, M., Musaya, J., Juziwelo, L. T., & Furu, P. (2022). An assessment of implementation and effectiveness of mass drug administration for prevention and control of schistosomiasis and soil-transmitted helminths in selected southern Malawi districts. BMC health services research, 22(1), 517.* [*https://doi.org/10.1186/s12913-022-07925-3*](https://doi.org/10.1186/s12913-022-07925-3)

*Asfaw MA, Hailu C, Beyene TJ. Evaluating Equity and Coverage in Mass Drug Administration for Soil-Transmitted Helminth Infections among School-Age Children in the Hard-to-Reach Setting of Southern Ethiopia. Pediatric Health Med Ther. 2021 Jul 8;12:325-333. doi: 10.2147/PHMT.S316194. PMID: 34267576; PMCID: PMC8275865.*

*World Health Organization. Guideline: Preventive Chemotherapy to Control Soil-Transmitted Helminth Infections in At-Risk Population Groups. World Health Organization; 2017. [PubMed] [Google Scholar]*

5. Authors could do more by briefly highlighting the roles of health workers in the entire MDA process. They only mentioned health workers play a crucial role, without highlighting those roles. This would be useful for readers not familiar with MDA process.

Thank you for your valuable input. We have added the roles of health workers.

“*Health workers facilitate MDA by distributing medication, educating communities, and monitoring progress to control and eliminate diseases effectively. Therefore, the health worker’s perceptions will provide insights into the challenges and enablers of the MDA control programs and significantly improve the programs, thus aiding in achieving the NTD 2030 goals [21-23]”* (See lines 99-103)

6. Can the authors provide a flow chart on how they performed these selections, since several criteria and steps were involved in the selection of these stakeholders? Your flow chart can also show the number of stakeholders available at each level, and how you have sampled them (selected a few of them) etc.

Thank you for your recommendation. The flow chart below showing the number of stakeholders and the number sampled has been added as Figure 1 (Flow Chart of Stakeholder’s Selection) (See line 157).

7. You can re-draw your table 3 without having so much rows, but instead columns, merge frequency and % in a column, and also, make the Roles as column headers.

Thank you for your feedback. The table has been modified, See line 131.
